# Inspiratory muscle training in patients with obesity: a systematic review and meta-analysis

**DOI:** 10.3389/fmed.2023.1284689

**Published:** 2023-11-27

**Authors:** Saúl Caicedo-Trujillo, Rodrigo Torres-Castro, Luis Vasconcello-Castillo, Lilian Solis-Navarro, Diana Sanchez-Ramirez, Rodrigo Núñez-Cortés, Roberto Vera-Uribe, Isabel Muñoz-Muñoz, Marisol Barros-Poblete, Juan Eduardo Romero, Jordi Vilaró

**Affiliations:** ^1^Centro Médico Familiar Integral y Especialidades Diálisis “LA MARISCAL”, Instituto Ecuatoriano de Seguridad Social (IESS), Quito, Ecuador; ^2^Programa de Doctorat-Facultat Ciències de la Salut Blanquerna, Universitat Ramon Llull, Barcelona, Spain; ^3^Department of Physical Therapy, Faculty of Medicine, University of Chile, Santiago, Chile; ^4^Department of Pulmonary Medicine, Hospital Clínic–Institut d’Investigacions Biomèdiques August Pi i Sunyer (IDIBAPS), University of Barcelona, Barcelona, Spain; ^5^Blanquerna School of Health Sciences, Global Research on Wellbeing (GRoW), Universitat Ramon Llull, Barcelona, Spain; ^6^Department of Respiratory Therapy, College of Rehabilitation Sciences, University of Manitoba, Winnipeg, MB, Canada; ^7^Physiotherapy in Motion Multispeciality Research Group (PTinMOTION), Department of Physiotherapy, University of Valencia, Valencia, Spain; ^8^Sección de Oncología, Hospital Clínico de la Universidad de Chile, Santiago, Chile; ^9^Programa de Doctorado en Ciencias Médicas, Universidad Austral de Chile, Valdivia, Chile

**Keywords:** inspiratory muscle training, obesity, physical capacity, maximal inspiratory pressure, lung function

## Abstract

**Introduction:**

Obesity is a chronic medical condition that affects, among others, the cardiovascular and respiratory systems. Interventions for its treatment focus on sustained weight reduction and general health improvement, leaving respiratory management aside. Our objective was to determine the effects of inspiratory muscle training (IMT) in patients with obesity.

**Methods:**

A systematic review was performed in Embase, Cochrane Library (CENTRAL), CINAHL, Web of Science, and PubMed/MEDLINE on June 26, 2023. Randomized clinical trials (RCTs), and quasi-randomized clinical trials investigating the effects of IMT in people with obesity were included. Selected studies were screened by two independent reviewers who extracted data and assessed the quality of the evidence.

**Results:**

The initial search returned 705 potential studies were included. Ultimately, eight studies met the criteria for eligibility and were included in the review. IMT improves physical capacity [6-minute walk test (6MWT): 44.5 m, 95% CI: 30.5 to 58.5; *p* < 0.0001] and the strength of the inspiratory muscles [maximal inspiratory pressure (MIP): −28.4 cm H_2_O, 95% CI: −41.9 to −14.8; *p* < 0.0001] compared to the controls, without differences in the pulmonary function, body mass index (BMI) and metabolic parameters.

**Conclusion:**

Inspiratory muscle training improves physical capacity and inspiratory muscle strength without significant changes in lung function, BMI, and metabolic parameters.

**Systematic review registration:** PROSPERO, identifier CRD42023439625, https://www.crd.york.ac.uk/prospero/display_record.php?ID=CRD42023439625.

## Introduction

Obesity is a chronic medical condition characterized by excessive accumulation of body fat, leading to adverse health outcomes (1). It is a major global health concern, increasing worldwide prevalence (2). According to the World Health Organization (WHO), the number of individuals with obesity has nearly tripled since 1975 (3). In 2016, the global prevalence of overweight and obesity was indeed significant. More than 1.9 billion adults were classified as overweight, and over 650 million were classified as obese (3). These alarming statistics highlight the urgent need for effective interventions to combat obesity and its associated health risks.

Obesity not only affects the body’s metabolic and cardiovascular systems but also places a significant burden on the respiratory system (4, 5). Excess weight can lead to decreased lung volumes, reduced lung compliance, and increased work of breathing (4, 6). It can also lead to the development or exacerbation of respiratory conditions such as obstructive sleep apnea, obesity, hypoventilation syndrome, and asthma (7–9). The compromised respiratory function in individuals with obesity contributes to impaired exercise tolerance, increased breathlessness, and a higher risk of respiratory complications (5, 10).

The treatment of obesity involves a multidisciplinary approach to achieve sustained weight loss and improve overall health (11). Pharmacological interventions, such as orlistat and liraglutide, may be prescribed to diminishing weight by reducing appetite or inhibiting fat absorption (12). Surgical interventions, especially bariatric surgery, are considered for individuals with severe obesity or obesity-related comorbidities (13). Dietary interventions, including calorie restriction and balanced macronutrient distribution, are crucial in weight management (14). Additionally, regular physical activity and exercise are essential components of obesity treatment, promoting energy expenditure and maintaining muscle mass (15). Specific exercises that target the respiratory muscles, such as inspiratory muscle training (IMT), have shown potential benefits in improving respiratory function in individuals with obesity (16, 17).

In addition to its potential role in obesity, respiratory muscle training has been explored as an alternative therapeutic option for various respiratory, cardiac, and metabolic conditions (18–20). It has been shown to improve respiratory muscle strength, endurance, and breathing efficiency, enhancing exercise capacity and quality of life (20, 21). Patients with chronic obstructive pulmonary disease, heart failure, and metabolic disorders have experienced significant benefits from respiratory muscle training (18, 20, 21).

Although IMT has demonstrated positive effects in managing various chronic conditions, its effectiveness in obesity is not yet fully understood. Given the compromised respiratory function in individuals with obesity and the potential benefits of respiratory muscle training, it is critical to investigate the effectiveness of respiratory muscle training in subjects with obesity is crucial. By determining the effects of such training on respiratory muscle strength, pulmonary function, exercise tolerance, and overall health outcomes, we can gain valuable insights into the potential benefits of this intervention in managing individuals with obesity.

## Methods

### Protocol and registration

A systematic review was performed following the Preferred Reporting Items for Systematic Reviews and Meta-Analyses (PRISMA) guidelines (22). The review was registered in the International Prospective Register of Systematic Reviews (PROSPERO) CRD42023439625.

### Criteria for considering studies in this review

Randomized clinical trials (RCTs), and quasi-randomized clinical trials, that investigated the effects of IMT in people with obesity were included. The studies should report on respiratory muscle strength, physical capacity, pulmonary function, or quality of life. The search approach was formulated using the PICO framework (population: people with obesity; intervention: IMT; control: no intervention; and outcome: physical capacity, pulmonary function, quality of life). All observational studies (retrospective, prospective, cross-sectional, longitudinal, case–control, and cohort), editorials, letters, conference publications, review articles, systematic reviews, meta-analyses, and *in vivo* and *in vitro* studies were excluded.

### Search strategies and data resources

Records were retrieved from Embase, Cochrane Library (CENTRAL), CINAHL, Web of Science, and PubMed/MEDLINE databases on June 26, 2023. Manual searches with the following terms: [(Obesity) OR (Obese)] AND [(Inspiratory muscle training) OR (Breathing exercises) OR (respiratory muscle training)] AND [(Pulmonary function) OR (Spirometry) OR (Maximal inspiratory pressure) OR (Maximal expiratory pressure) OR (MIP) OR (MEP) OR (Physical capacity) OR (6MWT) OR (CPET) OR (cardiopulmonary exercise test) OR (Dyspnea) OR (Fatigue) OR (Weight loss) OR (BMI)] were conducted. No language or publication restrictions were imposed. A manual search of the references list of the selected studies was also conducted. All references were analyzed using Rayyan web software (23).

### Reviewing procedure and data extraction

Two researchers with experience in meta-analysis and training in literature review independently assessed the articles. Initially, two investigators reviewed the titles and abstracts of all identified studies (SCT-LSN). Studies that were considered irrelevant based on the title and abstract review were excluded. In case of any disagreements, a third reviewer (RTC) resolved them. Subsequently, the full-text versions of the articles selected in the initial step were thoroughly examined against the eligibility criteria by the same reviewers (RTC-LSN). Any further discrepancies were addressed by involving a third reviewer (JV). Finally, additional unpublished data were obtained from study authors when possible. Two review authors extracted the data independently (LV-RTC). A third reviewer (JV) solved any disagreements in data extraction.

### Methodological quality assessment

The methodological quality of the included articles was evaluated using the quality assessment tools provided by the National Heart, Lung, and Blood Institute (NHLBI) (24). Each tool contains criteria for evaluating internal validity and risk of bias. The criteria were assessed using categories of “yes,” “no,” or “other” (indicating items like “not reported,” “not applicable,” or “not determinable”). An overall rating was assigned to each study, considering the items with affirmative answers: ≥ 75% was categorized as good, 50–75% as fair, and < 50% as poor. This evaluation was performed independently by two authors (SCT-LSN), and discrepancies were resolved through consensus. If disagreements could not be resolved, a third author (RTC) was consulted. Additionally, the overall certainty of the evidence was assessed independently by two reviewers (RTC, LSN) using the GRADE approach (25). Disagreements were solved by consensus. Publication bias was assessed by visualizing a funnel plot and Begg’s and Egger’s tests for the possible existence of study bias using the Jamovi software (version 2.3) (26).

### Data synthesis and analysis

Information relating to author, country, study design, number, and characteristics of patients included was collected and summarized in Table 1, characteristics of IMT interventions were collected and summarized in Table 2. Summaries of the association between the outcomes for each study in terms of mean differences (MD) or standard mean differences (SMD) were reported using Review Manager 5 (RevMan, Copenhagen: The Nordic Cochrane Center, The Cochrane Collaboration, 2014). Absolute values and obtained combined measures of the effect of each primary outcome through meta-analysis with a random-effect model due to the expected heterogeneity between studies were calculated (27). Statistical heterogeneity was measured with the *I*^2^ statistic and classified as negligeable (*I*^2^ = 0−40%), moderate (*I*^2^ = 30–60%), substantial (*I*^2^ = 50–90%), or considerable (*I*^2^ = 75–100%) (27).

**Table 1:** Characteristics of included studies.

Author, year	Country	Population	Design	Group	Subjects (M/F)	Age (years)	BMI (kg/m^2^)
Barbalho-Moulim et al. (28)	Brazil	Patients underwent bariatric surgery	RCT	Intervention	*n* = 15 (15/0)	36.13 ± 8.1	41.6 ± 4.7
				Control	*n* = 17 (17/0)	34.8 ± 9.5	42.1 ± 3
Casali et al. (29)	Brazil	Patients underwent bariatric surgery	RCT	Intervention	*n* = 15 (11/4)	37.6 ± 10.9	42.8 ± 4.2
				Control	*n* = 15 (11/4)	35.1 ± 10.7	43.6 ± 3.9
Tenório et al. (30)	Brazil	People with obesity	RCT	Intervention	*n* = 7 (NR)	37.9 ± 9.3	45.6 ± 4.7
				Control	*n* = 7 (NR)	33.4 ± 8.5	44.9 ± 3.6
Lloréns et al. (31)	Spain	Patients underwent bariatric surgery	RCT	Intervention	*n* = 23 (11/12)	43.7 ± 9.1	47.5 ± 4.3
				Control	*n* = 21 (12/9)	43.2 ± 10.9	51.6 ± 6.9
Edwards et al. (32)	Australia	People with obesity	MCT	Intervention	*n* = 35 (19/16)	46 ± 7.5	36.8 ± 7.4
				Control	*n* = 32 (18/14)	48 ± 11	35.2 ± 5.9
Ahmad et al. (33)	Egypt	People with DM2 and obesity	CT	Intervention	*n* = 14 (0/14)	42.2 ± 6	34.6 ± 4.6
				Control	*n* = 14 (0/14)	44.3 ± 7	36.8 ± 5.7
Kuo et al. (34)	Taiwan	People with obesity	MCT	Intervention	*n* = 16 (3/13)	37.6 ± 8.8	30.5 ± 2.8
				Control	*n* = 12 (4/8)	37.5 ± 8.5	31.1 ± 3.1
Kaeotawee et al. (17)	Thailand	Children and adolescents with obesity	RCT	Intervention	*n* = 20 (13/7)	12.4 ± 1.9	33.9 ± 5.5
				IS	*n* = 20 (12/8)	12.5 ± 2.6	30.9 ± 7.1
				Control	*n* = 20 (17/3)	11.2 ± 2.6	31.3 ± 5.8

BMI, Body mass index; COPD, Chronic obstructive pulmonary disease; CT, Clinical trial; DM2, Diabetes mellitus type 2; F, Female; IMT, Inspiratory muscle training; IS, Incentive spirometer; M, Male; MCT, Matched clinical trial; RCT, Randomized control trial; NR, Not reported; and y, years. Data are shown as Mean ± SD.

**Table 2:** Characteristics of interventions.

Study	Intervention				Control	Outcomes
	Device	Load	Time/Frequency	Progression		
Barbalho-Moulim et al. (28)	Threshold IMT Respironics, Pittsburgh, PA, United States	30% MIP	15 min daily. Six times/week. 2–4 weeks before surgery	At each visit to the physiotherapist (twice a week)	No intervention	RMS, lung volumes, and diaphragmatic excursion
Casali et al. (29)	Threshold IMT Respironics, Pittsburgh, PA, United States	40% MIP	Daily from 2nd to the 30th postoperative day. 20 min during hospital stay, and 30 min after discharge	Re-assessed MIP the 2nd, 7th, 14th, and 30th postoperative days	Sham (0 cm H_2_O)	RMS, pulmonary function, and inspiratory muscle endurance
Tenório et al. (30)	Threshold IMT Respironics, Pittsburgh, PA, United States	30% MIP	15 min (15–20 breaths per min)/twice daily/5 days a week for 12 weeks	MIP re-assessed weekly	No intervention	RMS, pulmonary function, respiratory muscle endurance, and diaphragmatic excursion
Lloréns et al. (31)	Threshold IMT Respironics, Pittsburgh, PA, United States	30% MIP	20 min daily/32–34 days before surgery	Progressively, daily when the Borg scale <5	Preoperative usual care	RMS, pulmonary function, and post-surgery oxygenation
Edwards et al. (32)	Powerbreathe (Powerbreathe International Ltd., Southam, United Kingdom)	55% MIP	Two sets daily of 30 reps for 4 weeks	Re-assessed MIP after 4 weeks of intervention	Placebo 10% MIP	RMS, pulmonary function, and functional exercise capacity
Ahmad et al. (33)	Threshold IMT Respironics, Pittsburgh, PA, United States	30% MIP	Once daily,10–25 min, 5 days per week for 8 weeks	Weeks 1–2: five sets, 20 reps/session. Weeks 3–5: five sets, 30 reps/session. Weeks 6–8: eight sets, 30 reps/session (2 min rest between sets)	Medical treatment	Health-related quality of life
Kuo et al. (34)	Powerbreathe (Powerbreathe International Ltd., Southam, United Kingdom)	55% MIP	Twice daily, 30 reps, 3 days a week for 6 weeks	Re-assessed after 6 weeks of intervention	Placebo 10% MIP	RMS, pulmonary function, functional exercise capacity, body composition, and blood lipids
Kaeotawee et al. (17)	Threshold IMT Respironics, Pittsburgh, PA, United States	40% MIP	Twice daily, three sets, 10 reps, a rest period of 1 min between each set for 8 weeks	NR	Control group NR	RMS, pulmonary function, and functional exercise capacity

MIP, Maximal inspiratory pressure; NA, Not applicable; NR, Not reported; RMS, Respiratory muscle strength.

## Results

### Study selection

**Figure 1:**
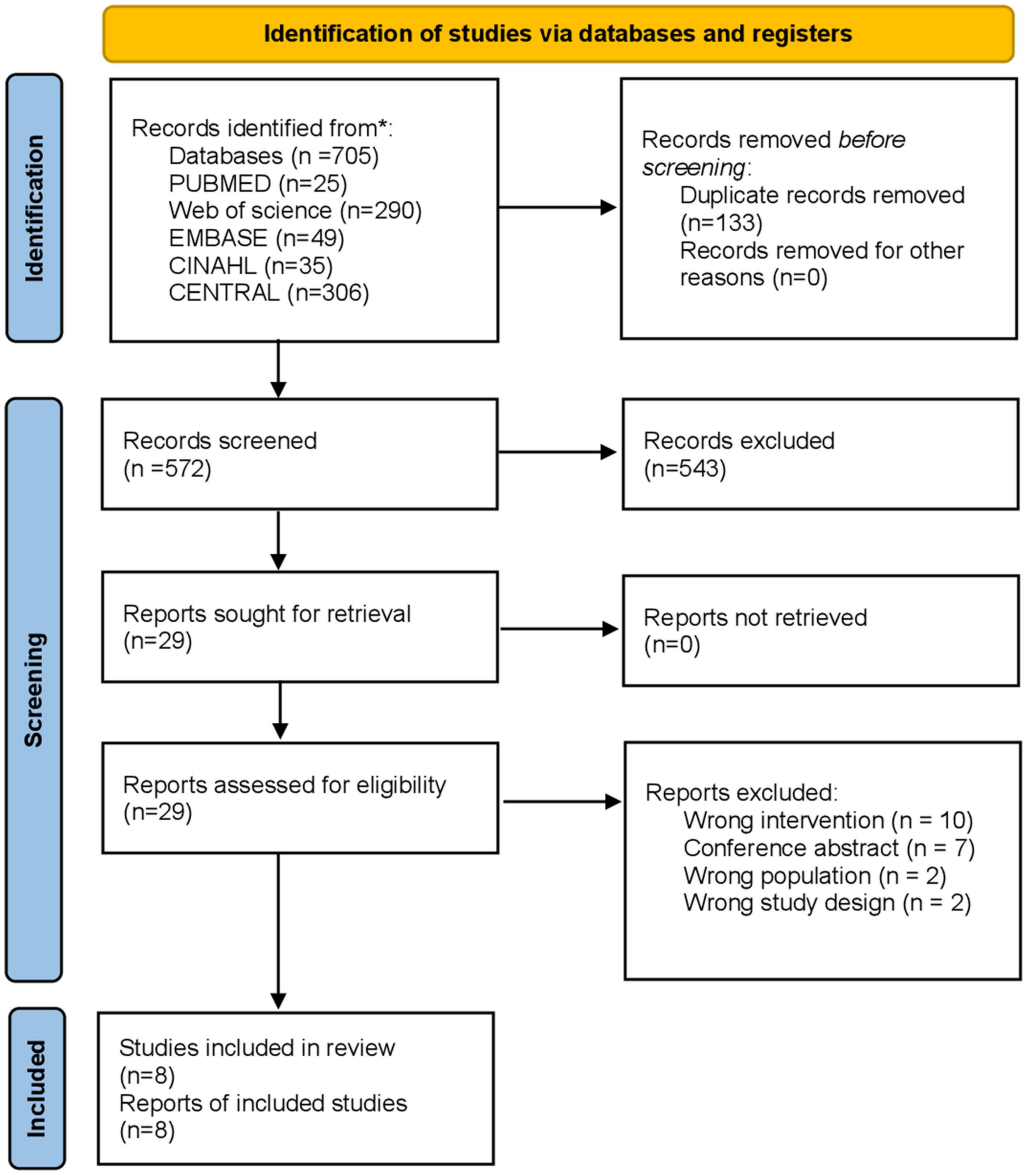
Study selection process.

The initial search yielded 705 potential studies. In total, 133 duplicate records were deleted. Five hundred seventy-two titles and abstracts were screened, and 543 records that did not meet our inclusion criteria were excluded. Twenty-nine of these were assessed as full-text. Of these, 10 were excluded for wrong intervention, seven for conference abstract, two for wrong population, and two for wrong study design. Ultimately, eight studies met the criteria for eligibility and were included in the review (17, 28–34). The flowchart of the study selection process is shown in Figure 1.

### Characteristics of the included studies

Three studies were conducted in Brazil (28–30), one in Spain (31), Australia (32), Egypt (33), Taiwan (34), and Thailand (17). All studies were published after 2011. Table 1 displays the attributes and features of the studies that have been incorporated in the analysis.

### Participants

A total of 283 participants with obesity were analyzed (145 in the intervention group and 138 in the control group). Sample sizes varied from 14 (30) to 67 (32) participants. The studies included 151 (56%) males and 118 (44%) females, with mean ages varying between 11.2 ± 2.6 (17) and 48 ± 11 (32) years. One study did not report the male/female distribution (30). The body mass index (BMI) varied between 30.5 ± 2.8 (34) and 51.6 ± 6.9 kg/m^2^ (Table 1) (31). The studies were conducted in adults (28–34), except one study that focused on children and adolescents (17). Additionally, some studies specifically targeted groups undergoing bariatric surgery (28, 29, 31), while others investigated important health conditions such as type 2 diabetes (33).

### Characteristics of training

The duration of training varied between 2 (28) and 12 weeks (30). The most commonly used load was 30% of maximal inspiratory pressure (MIP) (28, 30, 31, 33), two studies used 40% (17, 29), and two studies used loads of 55% (32, 34). Regarding the protocol, five studies were trained by time (28–31, 33), and three were trained by repetitions (17, 32, 34). The frequency varied from 3 (34) to 7 days per week (17, 28–32). The devices used were Powerbreathe (Powerbreathe, Southam, United Kingdom) (32, 34) or Threshold IMT (Respironics, Pittsburgh, PA, United States) (17, 28–31, 33). The control group received no intervention (28, 33), 10% MIP (32, 34), placebo (30), sham (29), preoperative usual care (31), or incentive spirometer (17).

### Methodological quality assessment

Only one of the RCT selected (12.5%) was rated as “good” (i.e., >75 affirmative answers), three (37.5%) were “fair” (50–75% affirmative answers), and four (50) were “poor” (<50% affirmative answers). The quality assessment results for the individual studies obtained using the NHLBI quality assessment tool are presented in the Supplementary material. In the estimation of the effect of IMT on MIP, the result of the Egger’s test suggests the presence of publication bias in the studies (*p* = 0.045), while the Begg’s test does not show solid evidence of such bias (*p* = 0.233).

### Main outcomes

#### Inspiratory muscle strength and endurance

**Figure 2:**
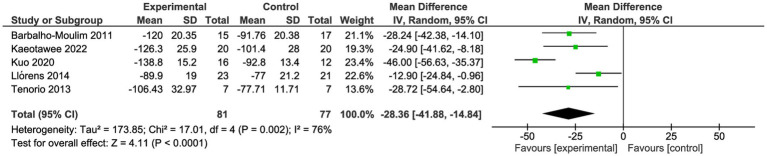
Forest plot for maximal inspiratory pressure.

Seven studies reported MIP (Table 2) (17, 28–32, 34). These studies compared 81 participants in the intervention group (IG) vs. 77 in the control group (CG). Patients in the IG had −28.4 cm H_2_O (95%CI -41.9 to −14.8) higher than CG (*p* < 0.0001; Figure 2). Substantial heterogeneity of the comparison was identified (*I*^2^ = 76%). We could only meta-analyze five studies (17, 28, 30, 31, 34); however, the two non-meta-analyzed studies reported increased MIP (29, 32). According to the GRADE methodology, the certainty of evidence was very low. Only one study reported on endurance, demonstrating that the intervention effectively improved it (29).

#### Physical capacity

**Figure 3:**
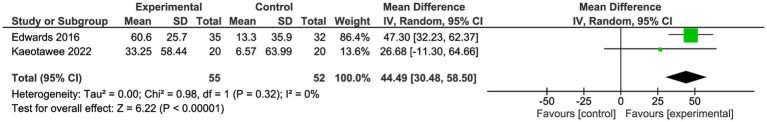
Forest plot for physical capacity.

Three studies reported physical capacity pre- and post-intervention (Table 2) (17, 32, 34). The outcome reported was the distance walked in the 6-min walk test (6MWD) (17, 32, 34). These studies compared 55 participants in the IG vs. 52 in the CG. Patients in the IG walked 44.5 m (95%CI 30.5–58.5) more compared to CG (*p* < 0.0001; Figure 3). The heterogeneity of the comparison was negligeable (*I*^2^ = 0%). The certainty of evidence, according to the GRADE methodology, was low.

#### Lung function

**Figure 4:**
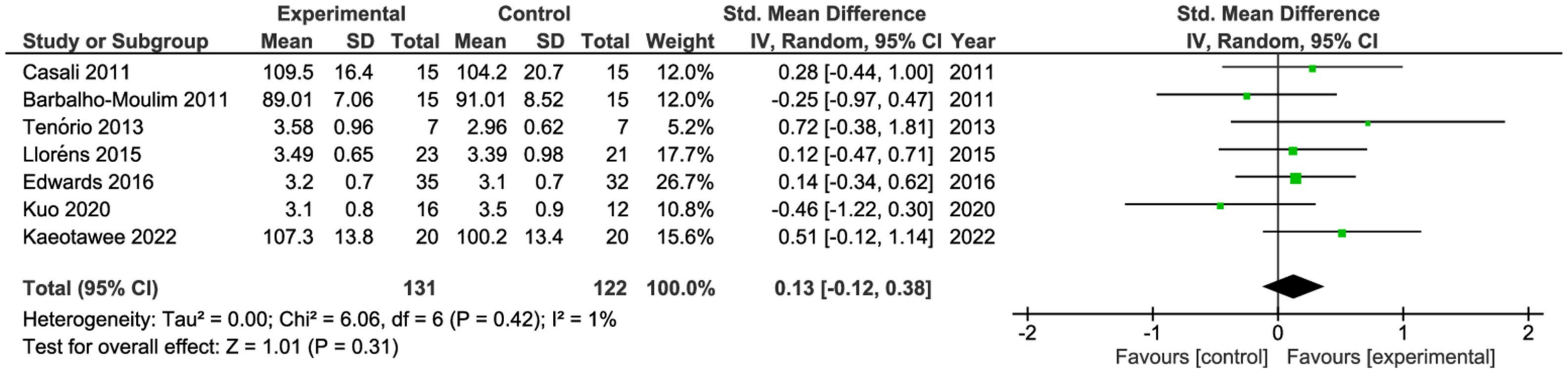
Forest plot for forced vital capacity.

**Figure 5:**
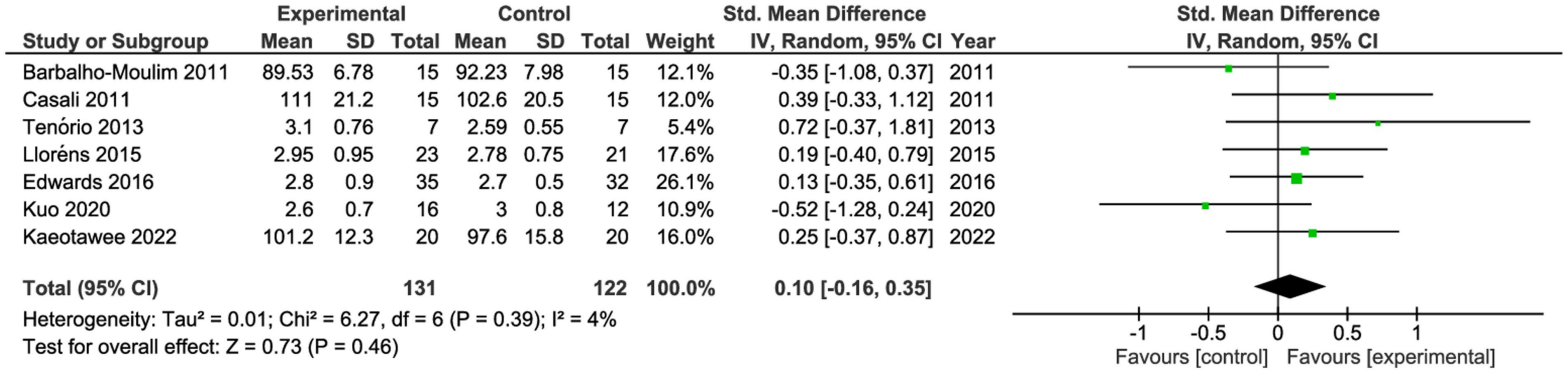
Forest plot for forced expiratory volume in the first second.

Seven studies reported lung function pre- and post-intervention (Table 2) (17, 28–32, 34). These studies compared 131 participants in the IG vs. 122 in the CG. Both groups had similar forced vital capacity (FVC; SMD 0.13; 95%CI −0.12 to 0.38, *p* = 0.31, *I*^2^: 1%) and similar forced expiratory volume in the first second (FEV_1_; SMD 0.10; 95%CI −0.16 to 0.35, *p* = 0.46, *I*^2^: 4%; Figures 4, 5). One study reported maximal voluntary ventilation with significant changes in IG (30). The certainty of evidence, according to the GRADE methodology, was low.

#### Body composition and metabolic parameters

Only one study assessed post-intervention BMI without finding significant differences after the intervention (34). The same authors also evaluated the lipid profile, finding no changes in cholesterol, triglycerides, high-density lipoprotein, or low-density lipoprotein (34). Another author assessed post-intervention glucose levels in 26 diabetic women who trained for 8 weeks at low intensity. They found no significant differences between the groups (33).

Additional outcomes reported pre- and post-intervention included quality of life (QoL) and dyspnea. Significant differences in QoL were found in a cohort of 26 diabetic women assessed with the SF-36 (33). On the other hand, no differences in post-exercise dyspnea were identified when assessed with the Borg scale (32).

## Discussion

Our main findings indicated that the IMT improves respiratory muscle strength and physical capacity without changing lung function in people with obesity.

Similarly to what has been observed in other conditions, IMT led to improvements in MIP since it targets the specific inspiratory muscles and utilizes flow-independent devices that adhere to training principles (35, 36). The suggested mechanisms contributing to this finding could involve an increase in the proportion and size of type II muscle fibers (34, 37, 38), promotion of diaphragm hypertrophy, attenuation of the respiratory muscle metaboreflex, and enhanced respiratory muscle economy (17). It is suggested that enhanced respiratory muscle strength increases respiratory capacity, facilitates muscle oxygenation, diminishes respiratory muscle fatigue, and improves individual performance for cardiopulmonary functioning during physical activity (28, 37, 39).

Physical capacity improved in three reviewed studies (17, 32, 34), although only two could be meta-analyzed due to their small sample size (17, 34). The improvement of respiratory muscle function directly impacts physical capacity since it partly depends on ventilation (40). By enhancing ventilatory capacity through the strength of respiratory muscles, patients can walk more while ventilating the same or less (40). Improved respiratory muscle strength enhances respiratory capacity, facilitates muscle oxygenation, lowers lactate production by respiratory muscles, and ultimately decreases respiratory muscle fatigue, improving exercise functional fitness (37, 39, 41). From a clinical point of view, despite the small sample sizes studied, the improvements exceeded the Minimal Clinically Important Difference (MCID) of 30 m in the 6MWT reported for patients with respiratory and cardiovascular diseases, suggesting that this intervention is effective for this outcome (42, 43).

Lung function remained unchanged, as seen in other conditions (19, 44). It is essential to highlight that this outcome can be of interest, especially considering that patients with obesity may develop a restrictive pattern (4). On the other hand, only one study evaluated maximal voluntary ventilation (MVV) and showed improvement suggesting that this intervention could be effective for this outcome (30). Although this test measures ventilation, it has been described in the literature as an assessment scale for the endurance of respiratory muscles (45).

Contrary to expectations, only one study reported variables related to body composition or metabolic parameters linked to obesity which are essential components to include in evaluating any patient with obesity and found no post-intervention changes (34). Similarly to some studies that have shown improvements in quality of life, only one of the analyzed studies found a significant improvement in QoL in subjects with obesity (33).

Three studies included patients eligible for bariatric surgery, making this potential group of interest. Lloréns et al. (31) reported that preoperative IMT enhanced postoperative oxygenation and raised inspiratory muscular strength among morbidly obese patients who underwent laparoscopic bariatric surgery. Similarly, Casali et al. (29) observed that IMT improves inspiratory muscle strength and endurance and accounts for earlier recovery of pulmonary airflows in morbidly obese patients submitted to bariatric surgery. Both studies confirm the improvement in post-surgical outcomes, suggesting the use of IMT in this subgroup of individuals.

### Limitations

Our study has some limitations. First, the selected studies are few and do not allow for a sub-analysis according to obesity severity or training loads. Second, a common feature of the studies is their small sample sizes. Third, regarding the interventions, the intensities and the time/frequency used in the included studies are so heterogeneous and provably low or short to achieve higher inspiratory muscle strength improvements. Fourth, the methodology quality is generally poor to consider the obtained evidence so strong to extrapolate their results to an obese general population. Finally, in the case of MIP, the Egger’s test’s result suggests publication bias in the studies, while the Begg’s test does not show solid evidence of such bias. In these cases, the validity and reliability of the meta-analysis should be considered with caution, as the presence of publication bias can affect the results and conclusions of the study.

## Conclusion

Inspiratory muscle training improves physical capacity, inspiratory muscle strength without significant changes in lung function, BMI, and metabolic parameters. Further studies are needed to improve the quality of the present evidence.

## Data availability statement

The original contributions presented in the study are included in the article/Supplementary material, further inquiries can be directed to the corresponding author.

## Author contributions

SC-T: Conceptualization, Data curation, Formal analysis, Investigation, Methodology, Writing – original draft, Writing – review & editing. RT-C: Conceptualization, Data curation, Formal analysis, Investigation, Methodology, Project administration, Software, Supervision, Validation, Visualization, Writing – original draft, Writing – review & editing. LV-C: Formal analysis, Investigation, Methodology, Software, Supervision, Writing – original draft, Writing – review & editing. LS-N: Conceptualization, Investigation, Methodology, Validation, Writing – original draft, Writing – review & editing. DS-R: Investigation, Validation, Writing – review & editing. RN-C: Conceptualization, Formal analysis, Methodology, Supervision, Validation, Writing – review & editing. RV-U: Conceptualization, Methodology, Validation, Writing – review & editing. IM-M: Data curation, Validation, Visualization, Writing – review & editing. MB-P: Formal analysis, Investigation, Writing – review & editing. JER: Methodology, Supervision, Writing – review & editing. JV: Conceptualization, Investigation, Methodology, Supervision, Validation, Visualization, Writing – original draft, Writing – review & editing.
