# Supplementary material for: Inspiratory muscle training in patients with obesity: a systematic review and meta-analysis
Source: Front Med (Lausanne). 2023 Nov 27;10:1284689. doi: 10.3389/fmed.2023.1284689 (PMC10711597; doi:10.3389/fmed.2023.1284689)
Supplement: Supplementary file 1 [file Table_1.DOCX]

| **Table S1**. Quality Assessment of Controlled Intervention Studies | | | | | | | | | | | | | | | | | |
| --- | --- | --- | --- | --- | --- | --- | --- | --- | --- | --- | --- | --- | --- | --- | --- | --- | --- |
| **Author** | **Year** | **1** | **2** | **3** | **4** | **5** | **6** | **7** | **8** | **9** | **10** | **11** | **12** | **13** | **14** | **Total Score** | **Quality Rating** |
| Ahmad et al | 2020 | N | NR | NR | NR | NR | Y | Y | Y | Y | Y | Y | NR | N | N | 6/14 | Poor |
| Barbalho et al | 2011 | Y | Y | Y | NR | NR | Y | NR | NR | NR | Y | Y | Y | Y | N | 8/14 | Fair |
| Casali et al | 2011 | N | NR | NR | NR | NR | Y | Y | NR | Y | Y | Y | N | N | N | 5/14 | Poor |
| Edwards et al | 2016 | N | NR | NR | NR | NR | Y | Y | NR | Y | Y | Y | N | N | N | 5/14 | Poor |
| Kaeotawee et al | 2022 | Y | Y | NR | NR | N | N | Y | Y | Y | Y | Y | Y | NR | Y | 9/14 | Fair |
| Kuo et al | 2020 | Y | N | NR | NR | N | Y | Y | Y | Y | Y | Y | N | Y | Y | 9/14 | Fair |
| Lloréns et al | 2015 | Y | Y | Y | Y | Y | N | Y | Y | Y | Y | Y | Y | N | Y | 12/14 | Good |
| Tenório et al | 2013 | N | NR | Y | Y | Y | NR | N | N | NR | NR | Y | NR | N | N | 4/14 | Poor |
| **Quality of included studies was assessed using the National Institutes of Health (NIH) Quality Assessment of Controlled Intervention Studies** (www.nhlbi.nih.gov/health-pro/guidelines/in-develop/cardiovascular-risk-reduction/tools/). **1**. Was the study described as randomized, a randomized trial, a randomized clinical trial, or an RCT? **2**. Was the method of randomization adequate (i.e., use of randomly generated assignment)? **3**. Was the treatment allocation concealed (so that assignments could not be predicted)? **4**. Were study participants and providers blinded to treatment group assignment? **5**. Were the people assessing the outcomes blinded to the participants' group assignments? **6**. Were the groups similar at baseline on important characteristics that could affect outcomes (e.g., demographics, risk factors, co-morbid conditions)? **7**. Was the overall drop-out rate from the study at endpoint 20% or lower of the number allocated to treatment? **8**. Was the differential drop-out rate (between treatment groups) at endpoint 15 percentage points or lower? **9**. Was there high adherence to the intervention protocols for each treatment group? **10**. Were other interventions avoided or similar in the groups (e.g., similar background treatments)? **11**. Were outcomes assessed using valid and reliable measures, implemented consistently across all study participants? **12**. Did the authors report that the sample size was sufficiently large to be able to detect a difference in the main outcome between groups with at least 80% power? **13**. Were outcomes reported or subgroups analyzed prespecified (i.e., identified before analyses were conducted)? **14**. Were all randomized participants analyzed in the group to which they were originally assigned, i.e., did they use an intention-to-treat analysis?  **Total Score:** Number of yes; **NR,** not reported; **N**, not present; **Y**, present.  **Quality Rating:** Poor <50%, Fair 50-75%, Good ≥75% | | | | | | | | | | | | | | | | | |
